# Supplementary material for: Youth, personality and collective victimhood distinguish support for radical climate action
Source: Commun Psychol. 2026 Feb 17;4:54. doi: 10.1038/s44271-026-00420-z (PMC13021965; doi:10.1038/s44271-026-00420-z)
Supplement: Supplementary file 2 — Supplementary Information [file 44271_2026_420_MOESM2_ESM.pdf]

Supplementary Information

Supplementary Methods

Table S1: Items, scale reliability indices, and sources

Concept	Wording and scale	Source of items
**DEMOGRAPHICS**		
Age	How old are you (enter age in years)?	
Gender	What is your gender? - Selected Choice<br><br>*1 = male, 2 = female, 3 = non-binary, 4 = prefer not to say, 5 = other, please specify ____*	
Education	What is the highest level of education you have completed?<br><br>*1 = did not graduate high school, 2 = completed high school or equivalent, 3 = certificate I-IV / TAFE, 4 = diploma or bachelor's degree, 5 = graduate certificate or graduate diploma, 6 = master's degree or doctoral degree* (participants also had the option to answer *would prefer not to say* which was treated as missing data)	
**INDIVIDUAL DIFFERENCES**		
Big 5 Personality<br><br>Extraverted + Reserved = extraversion (r = -.52) *95% CI* [.49, .55]<br><br>Critical + Warm = agreeableness (r = -.23) *95% CI* [-.19, -.27]<br><br>Dependable + Disorganised = conscientiousness (r = -.34) *95% CI* [-.30, -.38]	I see myself as ... - ... Extraverted, enthusiastic.<br><br>I see myself as ... - ... Critical, quarrelsome. (R)<br><br>I see myself as ... - ... Dependable, self-disciplined.<br><br>I see myself as ... - ... Anxious, easily upset.<br><br>I see myself as ... - ... Open to new experiences, complex.	Ten-item Personality Inventory: Gosling, S. D., Rentfrow, P. J. & Swann, W. B. A very brief measure of the Big-Five personality domains. *Journal of Research in Personality* **37**, 504–528 (2003).

Anxious + Calm = neuroticism ($r = -.53$) *95% CI* [-.49, -.55]<br><br>Open + Conventional = openness ($r = -.26$) *95% CI* [-.23, -.31]	I see myself as ... - ... Reserved, quiet. (R)<br><br>I see myself as ... - ... Sympathetic, warm.<br><br>I see myself as ... - ... Disorganised, careless. (R)<br><br>I see myself as ... - ... Calm, emotionally stable. (R)<br><br>I see myself as ... - ... Conventional, uncreative. (R)<br><br>*1 = strongly disagree, 2 = disagree a little, 3 = neither agree nor disagree, 4 = agree a little, 5 = agree strongly*	
Political conservatism	Politically speaking, would you describe yourself as generally left-wing (progressive) or right-wing (conservative)?<br><br>*1 (Left-Wing) - 7 (Right-Wing)*	
**CLIMATE-RELATED BELIEFS**		
Belief in climate change<br>($r = .77$) *95% CI* [.76, .79]	I believe that climate change is happening<br><br>I believe that present-day climate change is caused in part by human activities<br><br>*1 (strongly disagree) - 7 (strongly agree)*	
Moral conviction<br>($\alpha = .82$)	I believe that the issue of climate change is a moral issue.<br><br>My opinion on climate change is part of my core moral norms and values.<br><br>My opinion on climate change is deeply connected to my beliefs about “right” and “wrong”.<br><br>*1 (strongly disagree) - 7 (strongly agree)*	Adapted from Reifen Tagar, M., Morgan, G., Halperin, E., & Skitka, L. When ideology matters: Moral conviction and the association between ideology and policy preferences in the Israeli–Palestinian conflict. *European Journal of Social Psychology* **44**, 117-125 (2014).

**EMOTION**		
Anger	When I think about the current debates on climate change, I feel angry.<br><br>*1 (strongly disagree) - 7 (strongly agree)*	Thomas, E. F., & McGarty, C. The role of efficacy and moral outrage norms in creating the potential for international development activism through group-based interaction. *British Journal of Social Psychology* **48**, 115-134 (2009).
**BELIEFS ABOUT THE MOVEMENT**		
Identification	How much do you identify with other people who have similar views on climate change as you do?<br><br>*1 (not at all) - 7 (very much)*	Postmes, T., Haslam, S. A. & Jans, L. A single-item measure of social identification: Reliability, validity, and utility. *British Journal of Social Psychology* **52**, 597-617 (2013).
Collective efficacy<br>(r = .74) *95% CI* [.72, .76]	I think people concerned with climate change have the ability to reduce the impacts of climate change through unified action.<br><br>I think people concerned with climate change can create a big change in the political debate if they choose to.<br><br>*1 (strongly disagree) - 7 (strongly agree)*	
**INTERGROUP BELIEFS**		
Collective victimhood<br>(r = .77) *95% CI* [.75, .79]	Throughout the climate crisis conflict, supporters of climate change action have suffered more than opponents of climate change action.<br><br>In recent times, supporters of climate change action have suffered from the	Adapted from Noor, M., Shnabel, N., Halabi, S., & Nadler, A. When suffering begets suffering: The psychology of competitive victimhood between adversarial

	behaviour of opponents of climate change action.<br><br>*1 (strongly disagree) - 7 (strongly agree)*	groups in violent conflicts. *Personality and Social Psychology Review* **16**, 351-374 (2012).
State capture<br>(α = .92)	Fossil fuel and mining corporations have too much power over politicians.<br><br>Fossil fuel and mining corporations strategically hire ex-politicians as consultants or directors in order to motivate politicians to align with their interests while they are in office.<br><br>The institutions of government have been captured by fossil fuel and mining companies.<br><br>*1 (strongly disagree) - 7 (strongly agree)*	
Outgroup favourability	Please provide a number between 0° and 100°, to indicate your attitude toward people who hold the opposing view to you on climate change:<br><br>*0 (extremely unfavourable) - 100 (extremely favourable)*	Druckman, J.N. & Levendusky, M.S. What do we measure when we measure affective polarization? *Public Opinion Quarterly* **83**, 114–122 (2019).
Norms of intergroup hostility<br>(α = .90)	For the next questions, we are not asking about what you think, we are asking you to imagine what supporters of climate action think. In your view, how much do supporters of climate action think that…<br><br>People who do not take action on climate change are morally wrong.<br><br>People who do not take action on climate change cause pain and suffering.<br><br>People who do not take action on climate change deserve to be confronted.<br><br>People who do not take action on climate change deserve to be opposed.<br><br>*1 (not at all) - 7 (very much)*	
Empathy for outgroup<br>(r = .87) *95% CI* [.86, .88]	When I think about the current debates on climate change, I feel …	

	empathy for people with different views<br><br>compassion for people with different views<br><br>*1 (strongly disagree) - 7 (strongly agree)*	
**COLLECTIVE ACTION INTENTIONS**		
Conventional collective action intentions<br>(α = .89)	I intend to join an organisation that supports action on climate change.<br><br>I intend to donate money to an organisation that supports action on climate change.<br><br>I intend to volunteer my time working (i.e., writing petitions, distributing flyers, recruiting people, etc.) for an organization that supports action on climate change.<br><br>I intend to join a public rally, protest, or demonstration that supports action on climate change.<br><br>I intend to participate in a sit-in to call on leaders to take action on climate change.<br><br>I intend to boycott businesses that stand in the way of action on climate change.<br><br>I intend to support a politician who is willing to take action on climate change.<br><br>*1 (strongly disagree) - 7 (strongly agree)*	Adapted from the following paper to apply to the climate change context: Moskalenko, S., & McCauley, C. Measuring political mobilization: The distinction between activism and radicalism. *Terrorism and Political Violence* **21**, 239-260 (2009).
Radical collective action intentions<br>(α = .91)	I intend to donate to an organization that supports action on climate change but that sometimes breaks the law.<br><br>I intend to support an organization that supports action on climate change but that sometimes resorts to violence.<br><br>I intend to participate in a public protest to fight to take action on climate change even though it might turn violent.	Adapted from the following paper to apply to the climate change context: Moskalenko, S., & McCauley, C. Measuring political mobilization: The distinction between activism and radicalism. *Terrorism and Political Violence* **21**, 239-260 (2009).

	I intend to join protests involving chaining myself to fences to highlight the need for society to take action on climate change.
	I intend to vandalise businesses that stand in the way of action on climate change.
	I intend to participate in blockades of roads or motorways to highlight the need for action on climate change.
	1 (strongly disagree) - 7 (strongly agree)

Table S2a: Logistic regression predicting dropout from Wave 1 to Wave 2

Variable	*B*	95% CI	*SE*	*z*	*p*
**Age**	**-0.028**	[-0.035, -0.020]	**0.004**	**-7.010**	**< .001**
Gender	0.043	[-0.199, 0.286]	0.124	0.352	.725
Political conservatism	-0.034	[-0.129, 0.061]	0.049	-0.700	.484
Education	-0.078	[-0.166, 0.009]	0.045	-1.751	.080
Extraversion	0.132	[0.010, 0.253]	0.062	2.130	.033
Agreeableness	0.050	[-0.109, 0.210]	0.081	0.617	.538
Conscientiousness	-0.214	[-0.388, -0.040]	0.089	-2.410	.016
Neuroticism	-0.066	[-0.205, 0.074]	0.071	-0.922	.357
Openness	0.085	[-0.079, 0.249]	0.084	1.020	.308
**Belief in climate change**	**-0.239**	[-0.381, -0.097]	**0.072**	**-3.308**	**.001**
Moral conviction	-0.060	[-0.177, 0.057]	0.060	-1.001	.317
Identification	-0.157	[-0.274, -0.041]	0.059	-2.650	.008
Collective efficacy	-0.054	[-0.170, 0.061]	0.059	-0.920	.357
Empathy for outgroup	0.078	[-0.011, 0.167]	0.045	1.715	.086
Norm of hostility	0.084	[0.006, 0.162]	0.040	2.123	.034
State capture	-0.063	[-0.169, 0.042]	0.054	-1.176	.240
Collective victimhood	-0.091	[-0.190, 0.009]	0.051	-1.788	.074
Anger	0.100	[0.014, 0.186]	0.044	2.277	.023
Radical collective action	-0.061	[-0.215, 0.093]	0.079	-0.779	.436
Conventional collective action	0.023	[-0.118, 0.164]	0.072	0.324	.746

Table S2b: Logistic regression predicting dropout from Wave 2 to Wave 3

Variable	*B*	95% CI	*SE*	*z*	*p*
Age	-0.011	[-0.022, 0.001]	0.006	-1.803	.072
Gender	-0.083	[-0.419, 0.254]	0.172	-0.481	.631
Political conservatism	0.063	[-0.077, 0.203]	0.072	0.883	.377
Education	-0.044	[-0.169, 0.081]	0.064	-0.697	.486
Extraversion	0.108	[-0.066, 0.282]	0.089	1.216	.224
Agreeableness	-0.128	[-0.343, 0.087]	0.110	-1.167	.243
Conscientiousness	-0.196	[-0.454, 0.062]	0.132	-1.487	.137
Neuroticism	0.109	[-0.082, 0.299]	0.097	1.121	.262
Openness	0.216	[-0.014, 0.447]	0.118	1.837	.066
Belief in climate change	-0.120	[-0.325, 0.084]	0.104	-1.154	.248
Moral conviction	0.068	[-0.099, 0.236]	0.085	0.801	.423
Identification	-0.105	[-0.274, 0.063]	0.086	-1.223	.221
Collective efficacy	0.025	[-0.131, 0.181]	0.080	0.312	.755
Empathy for outgroup	-0.017	[-0.146, 0.112]	0.066	-0.255	.799
Norm of hostility	-0.120	[-0.247, 0.007]	0.065	-1.845	.065
State capture	-0.163	[-0.324, -0.003]	0.082	-1.996	.046
Collective victimhood	0.036	[-0.114, 0.186]	0.077	0.474	.635
Anger	0.115	[-0.003, 0.234]	0.060	1.908	.056

Radical collective action	-0.038	[-0.274, 0.198]	0.120	-0.314	.754
Conventional collective action	-0.072	[-0.273, 0.130]	0.103	-0.697	.486

Table S2c: Logistic regression predicting dropout from Wave 1 to Wave 3

**Variable**	***B***	**95% CI**	***SE***	***z***	***p***
**Age**	**-0.023**	[-0.031, -0.015]	**0.004**	**-5.832**	**< .001**
Gender	0.205	[-0.039, 0.449]	0.125	1.644	.100
Political conservatism	0.002	[-0.093, 0.097]	0.049	0.045	.964
Education	-0.063	[-0.151, 0.025]	0.045	-1.408	.159
Extraversion	0.080	[-0.042, 0.202]	0.062	1.280	.200
Agreeableness	-0.070	[-0.232, 0.091]	0.082	-0.857	.391
Conscientiousness	-0.270	[-0.450, -0.089]	0.092	-2.931	.003
Neuroticism	0.011	[-0.130, 0.152]	0.072	0.150	.881
Openness	0.217	[0.052, 0.383]	0.084	2.571	.010
Belief in climate change	-0.170	[-0.315, -0.024]	0.074	-2.286	.022
Moral conviction	-0.054	[-0.172, 0.063]	0.060	-0.908	.364
Identification	-0.125	[-0.243, -0.007]	0.060	-2.082	.037
Collective efficacy	-0.030	[-0.147, 0.087]	0.060	-0.506	.613
Empathy for outgroup	0.024	[-0.065, 0.114]	0.046	0.532	.595
Norm of hostility	0.078	[0.000, 0.157]	0.040	1.957	.050
State capture	-0.109	[-0.217, -0.002]	0.055	-1.999	.046
Collective victimhood	-0.078	[-0.178, 0.022]	0.051	-1.522	.128
Anger	0.122	[0.035, 0.208]	0.044	2.765	.006
Radical collective action	-0.139	[-0.294, 0.017]	0.079	-1.744	.081
Conventional collective action	0.038	[-0.103, 0.179]	0.072	0.526	.599

Note. Gender is coded such that *1 = male, 2 = female*. *SE* = standard error. Rows in bold are significant effects after applying a Bonferroni correction for multiple analyses ($p < .002$).

Table S3. Principal Components Analysis (with oblimin rotation) on collective action items

	Initial eigenvalues			Extraction sums of squared loadings		
	**Total**	**% of Variance**	**Cumulative %**	**Total**	**% of Variance**	**Cumulative %**
1	6.676	51.35	51.35	6.676	51.35	51.35
2	2.232	17.17	68.52	2.232	17.17	68.52
3	.864	6.65	75.17			
4	.613	4.72	79.89			
5	.486	3.74	83.63			
6	.378	2.91	86.53			
7	.342	2.63	89.16			
8	.296	2.28	91.44			
9	.274	2.11	93.55			
10	.241	1.85	95.40			
11	.228	1.76	97.16			
12	.188	1.45	98.60			
13	.182	1.40	100.00			

Pattern Matrix from Principal Components Analysis with Oblimin Rotation

Item wording	Component 1 (Radical)	Component 2 (Conventional)
I intend to join an organisation that supports action on climate change.	.205	**.731**
I intend to donate money to an organisation that supports action on climate change.	.040	**.791**
I intend to volunteer my time working (i.e., writing petitions, distributing flyers, recruiting people, etc.).	.185	**.746**

I intend to join a public rally, protest, or demonstration that supports action on climate change.	.226	**.732**
I intend to participate in a sit-in to call on leaders to take action on climate change.	.398	**.577**
I intend to boycott businesses that stand in the way of action on climate change.	–.082	**.796**
I intend to support a politician who is willing to take action on climate change.	–.288	**.790**
I intend to donate to an organization that supports action on climate change but that sometimes breaks the law.	**.514**	.429
I intend to support an organization that supports action on climate change but that sometimes resorts to violence.	**.807**	.043
I intend to participate in a public protest … even though it might turn violent.	**.840**	.087
I intend to join protests involving chaining myself to fences …	**.885**	.009
I intend to vandalise businesses that stand in the way of action on climate change.	**.914**	–.121
I intend to participate in blockades of roads or motorways …	**.865**	.021

Note. Extraction Method: Principal Component Analysis. Rotation Method: Oblimin with Kaiser Normalization. Rotation converged in 9 iterations.

Table S4. Means and standard deviations for each of the collective action items

Item wording	*N*	*M* (*SD*)
I intend to join an organisation that supports action on climate change.	1420	2.70 (1.51)
I intend to donate money to an organisation that supports action on climate change.	1417	3.20 (1.71)
I intend to volunteer my time working (i.e., writing petitions, distributing flyers, recruiting people, etc.).	1417	2.68 (1.56)
I intend to join a public rally, protest, or demonstration that supports action on climate change.	1418	2.63 (1.69)
I intend to participate in a sit-in to call on leaders to take action on climate change.	1410	2.16 (1.43)
I intend to boycott businesses that stand in the way of action on climate change.	1420	3.75 (1.88)
I intend to support a politician who is willing to take action on climate change.	1421	4.98 (1.66)
I intend to donate to an organization that supports action on climate change but that sometimes breaks the law.	1420	2.17 (1.50)
I intend to support an organization that supports action on climate change but that sometimes resorts to violence.	1419	1.67 (1.24)
I intend to participate in a public protest … even though it might turn violent.	1417	1.64 (1.19)
I intend to join protests involving chaining myself to fences …	1419	1.50 (1.09)
I intend to vandalise businesses that stand in the way of action on climate change.	1415	1.31 (0.94)
I intend to participate in blockades of roads or motorways …	1420	1.42 (1.03)

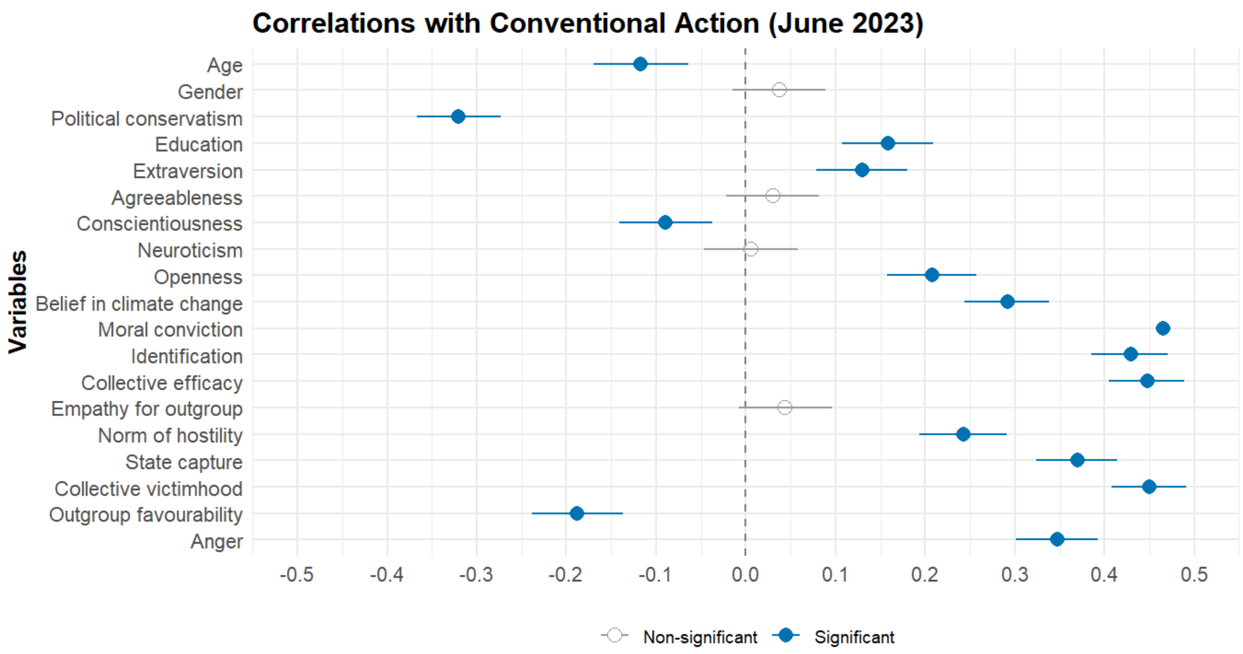

Figure S1: Correlations with conventional collective action intentions. Error bars are 95% confidence intervals. Shaded in diamonds represent significant correlations. *N* = 1,427 participants.

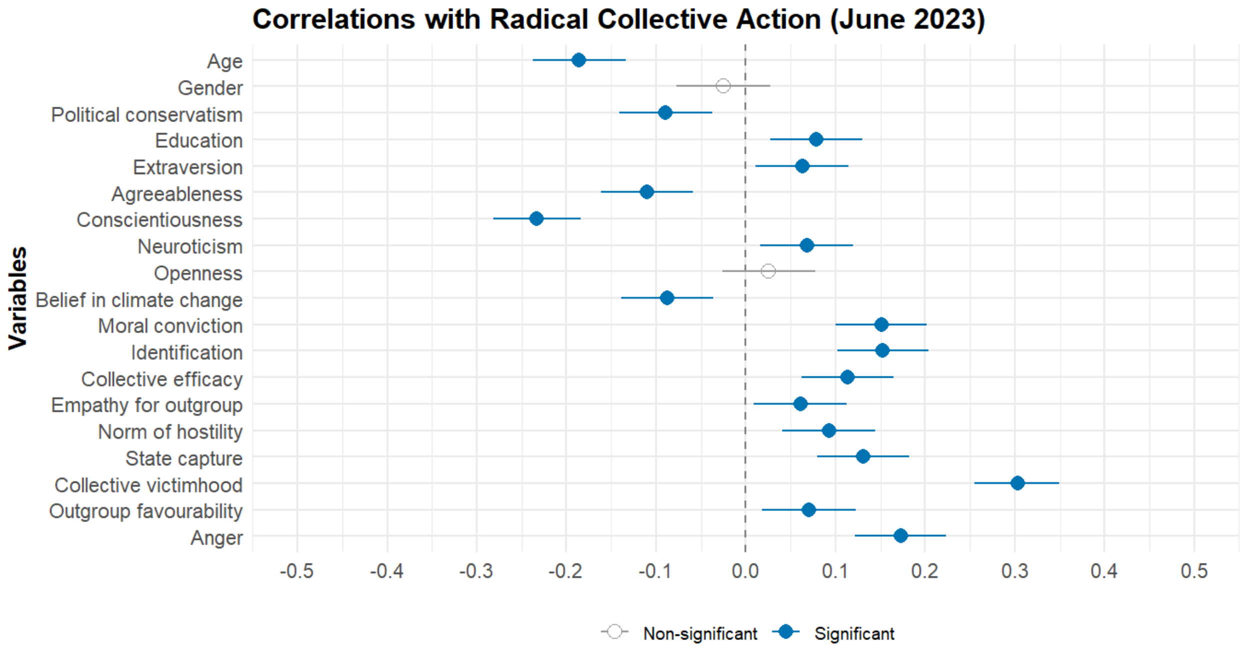

Figure S2: Correlations with radical collective action intentions. Error bars are 95% confidence intervals. Shaded in diamonds represent significant correlations. $N = 1{,}427$ participants.

Table S5

Cross-Lagged Associations Between Conventional Collective Action and Psychological Variables: Dynamic Structural Equation Model Results

Predictor Variable	Cross-Lagged Path	Estimate	95% Credibility Interval
**Anger ↔ CA**	Anger_{t-1} → CA_t	0.019	[-0.040, 0.078]
	CA_{t-1} → Anger_t	0.139	[0.061, 0.233]
**Moral Conviction ↔ CA**	Moral_{t-1} → CA_t	0.027	[-0.038, 0.093]
	CA_{t-1} → Moral_t	0.158	[0.086, 0.239]
**Identification↔ CA**	Identification_{t-1} → CA_t	0.096	[-0.002, 0.186]
	CA_{t-1} → Identification_t	0.410	[0.150, 0.665]
**Efficacy ↔ CA**	Efficacy_{t-1} → CA_t	0.088	[0.003, 0.176]
	CA_{t-1} → Efficacy_t	0.499	[0.366, 0.631]
**Empathy ↔ CA**	Empathy_{t-1} → CA_t	-0.047	[-0.100, 0.016]
	CA_{t-1} → Empathy_t	-0.066	[-0.147, 0.004]
**Collective Victimhood ↔ CA**	Collective Vict_{t-1} → CA_t	0.059	[-0.008, 0.128]
	CA_{t-1} → Collective Vict_t	0.196	[0.113, 0.306]

Note. CA = Collective Action. All estimates are unstandardized regression coefficients from multilevel dynamic structural equation models using Bayesian estimation. Credibility intervals are reported instead of traditional confidence intervals because Bayesian methods produce posterior probability distributions for parameters. A 95% credibility interval indicates there is a 95% probability that the true parameter value lies within the specified range, given the observed data and prior assumptions. The *p*-values represent one-tailed posterior probabilities that the parameter is greater than zero.

Table S6

Cross-Lagged Associations Between Radical Collective Action and Psychological Variables: Dynamic Structural Equation Model Results

Predictor Variable	Cross-Lagged Path	Estimate	95% Credibility Interval
**Anger ↔ RA**	Anger_{t-1} → RA_t	0.044	[-0.030, 0.115]
	RA_{t-1} → Anger_t	0.028	[-0.048, 0.117]
**Moral Conviction ↔ RA**	Moral_{t-1} → RA_t	0.033	[-0.038, 0.100]
	RA_{t-1} → Moral_t	0.015	[-0.064, 0.088]
**Identification ↔ RA**	Identification_{t-1} → RA_t	0.036	[-0.034, 0.105]
	RA_{t-1} → Identification_t	0.012	[-0.060, 0.080]
**Efficacy ↔ RA**	Efficacy_{t-1} → RA_t	0.082	[-0.062, 0.361]
	RA_{t-1} → Efficacy_t	0.136	[-0.028, 0.489]
**Empathy ↔ RA**	Empathy_{t-1} → RA_t	0.055	[-0.022, 0.149]
	RA_{t-1} → Empathy_t	0.026	[-0.058, 0.128]
**Collective Victimhood ↔ RA**	Collective Vict_{t-1} → RA_t	0.079	[-0.002, 0.188]
	RA_{t-1} → Collective Vict_t	0.070	[-0.027, 0.234]

Note. RA = Radical Collective Action. All estimates are standardized regression coefficients from multilevel dynamic structural equation models using Bayesian estimation. Credibility intervals are reported instead of traditional confidence intervals because Bayesian methods produce posterior probability distributions for parameters. A 95% credibility interval indicates there is a 95% probability that the true parameter value lies within the specified range, given the observed data and prior assumptions. The *p*-values represent one-tailed posterior probabilities that the parameter is greater than zero.

Figure S3. Cloud plots displaying distributions and central tendencies of the 19 predictors and 2 outcome variables.

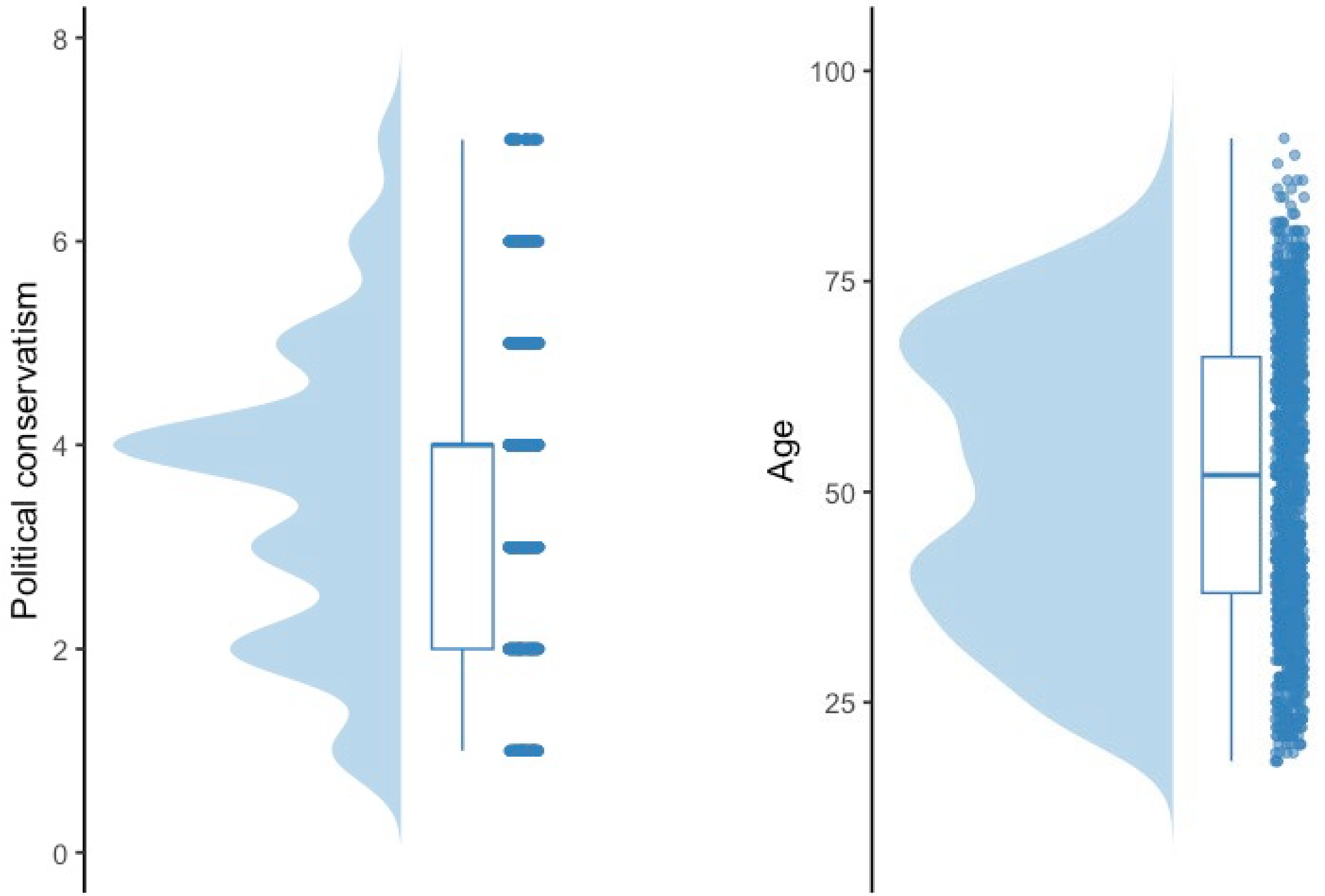

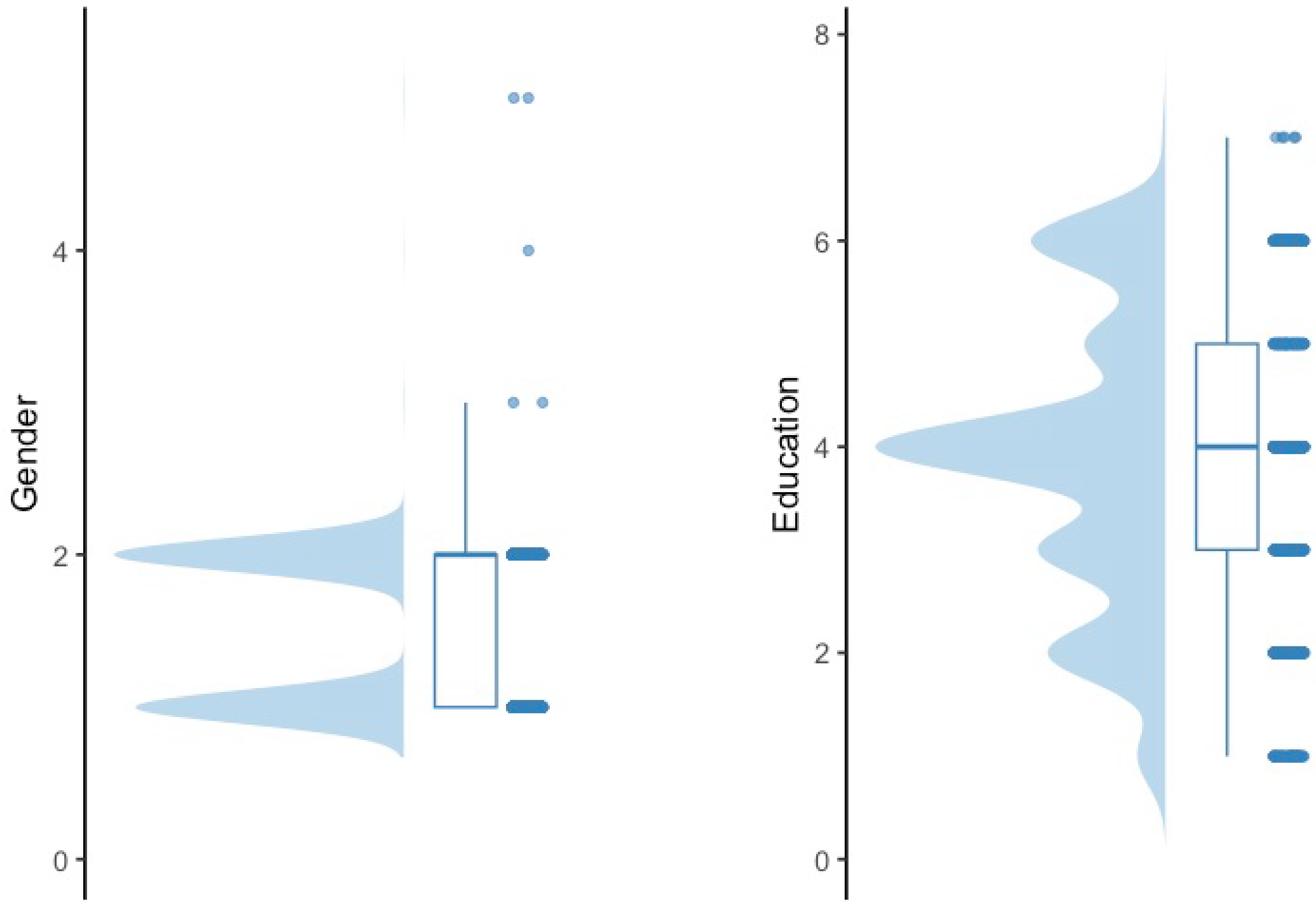
Gender
0
2
4
Education
0
2
4
6
8

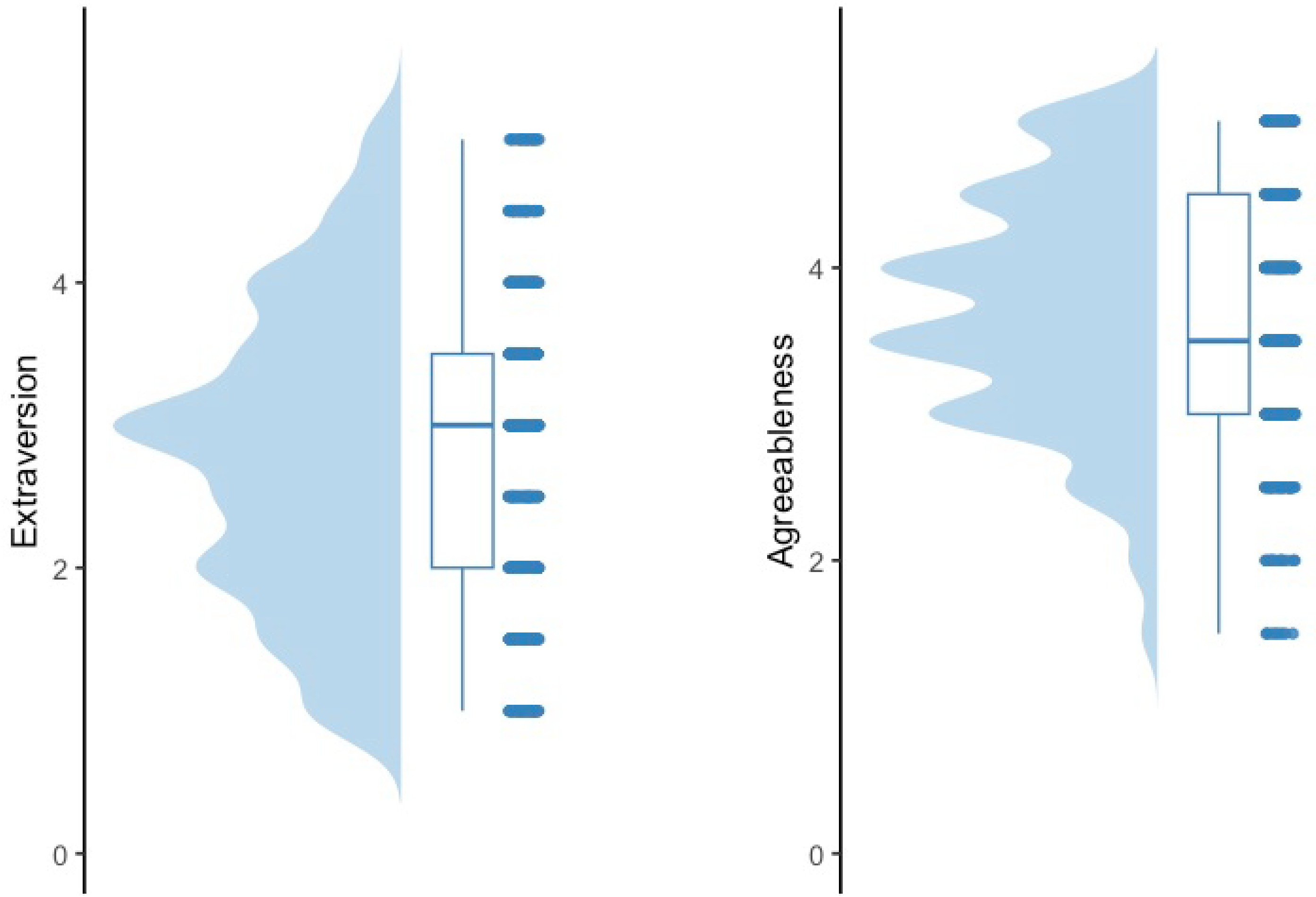
Extraversion
4
2
0
Agreeableness
4
2
0

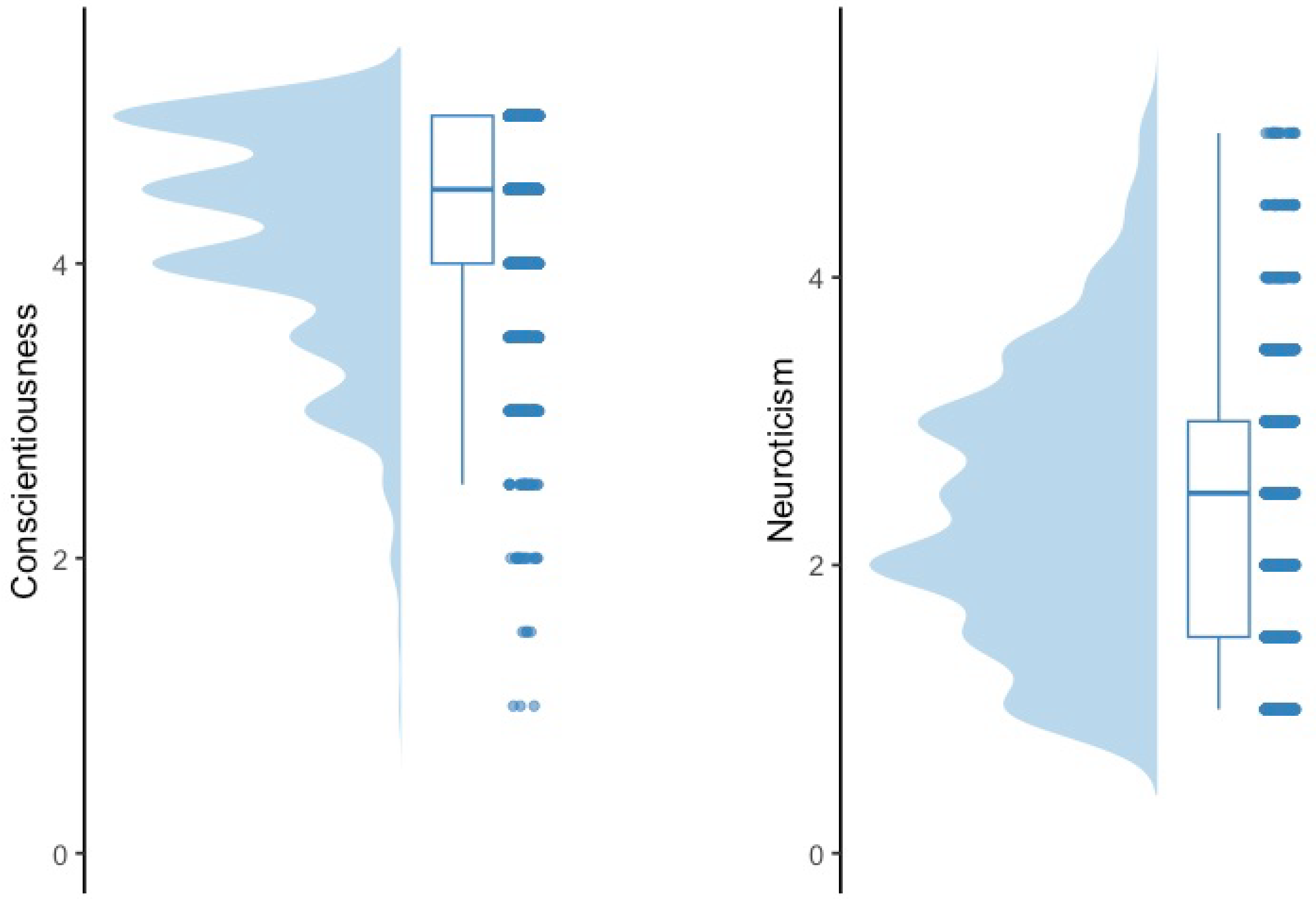

Conscientiousness
4
2
0
Neuroticism
4
2
0

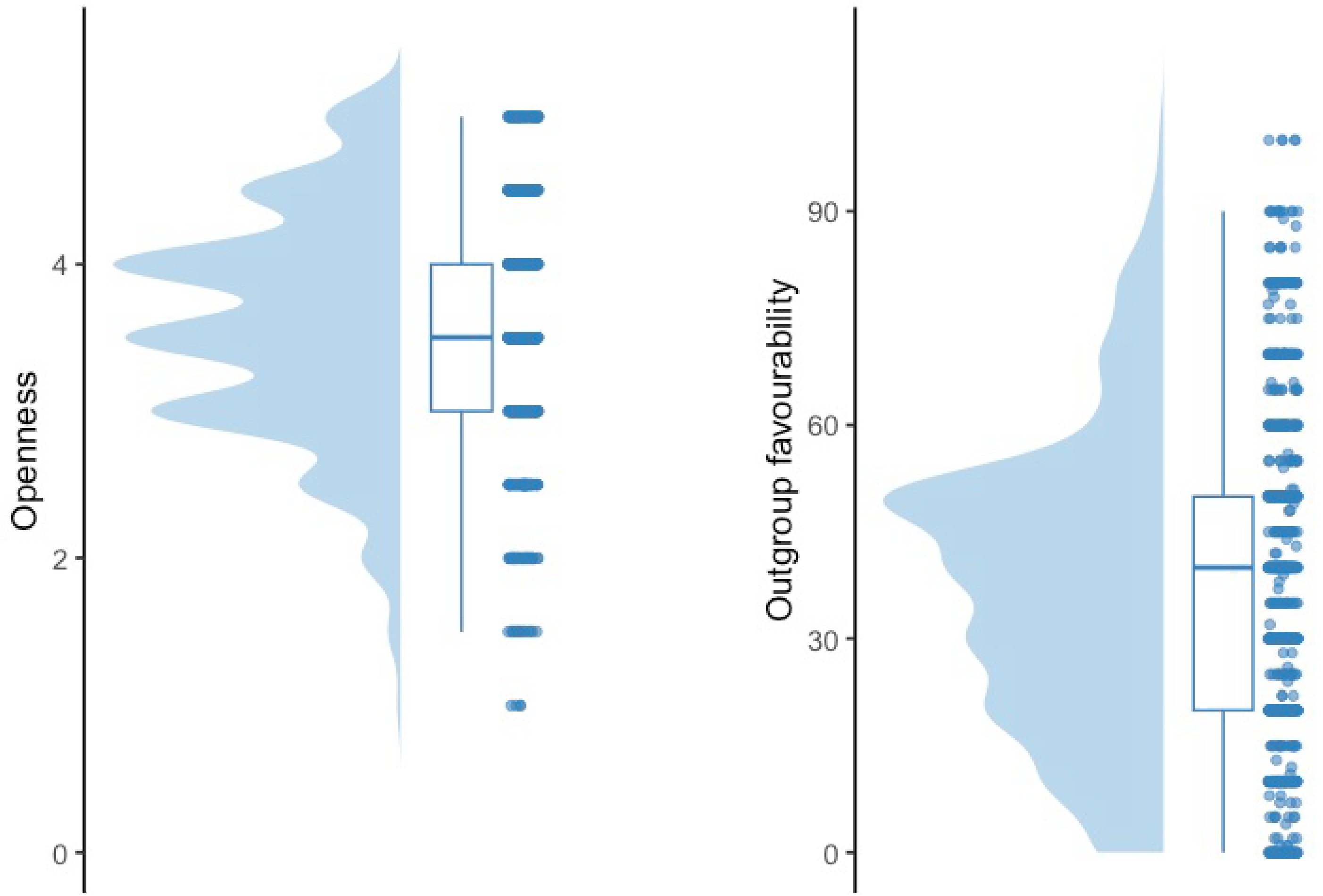

Openness
4
2
0
Outgroup favourability
90
60
30
0

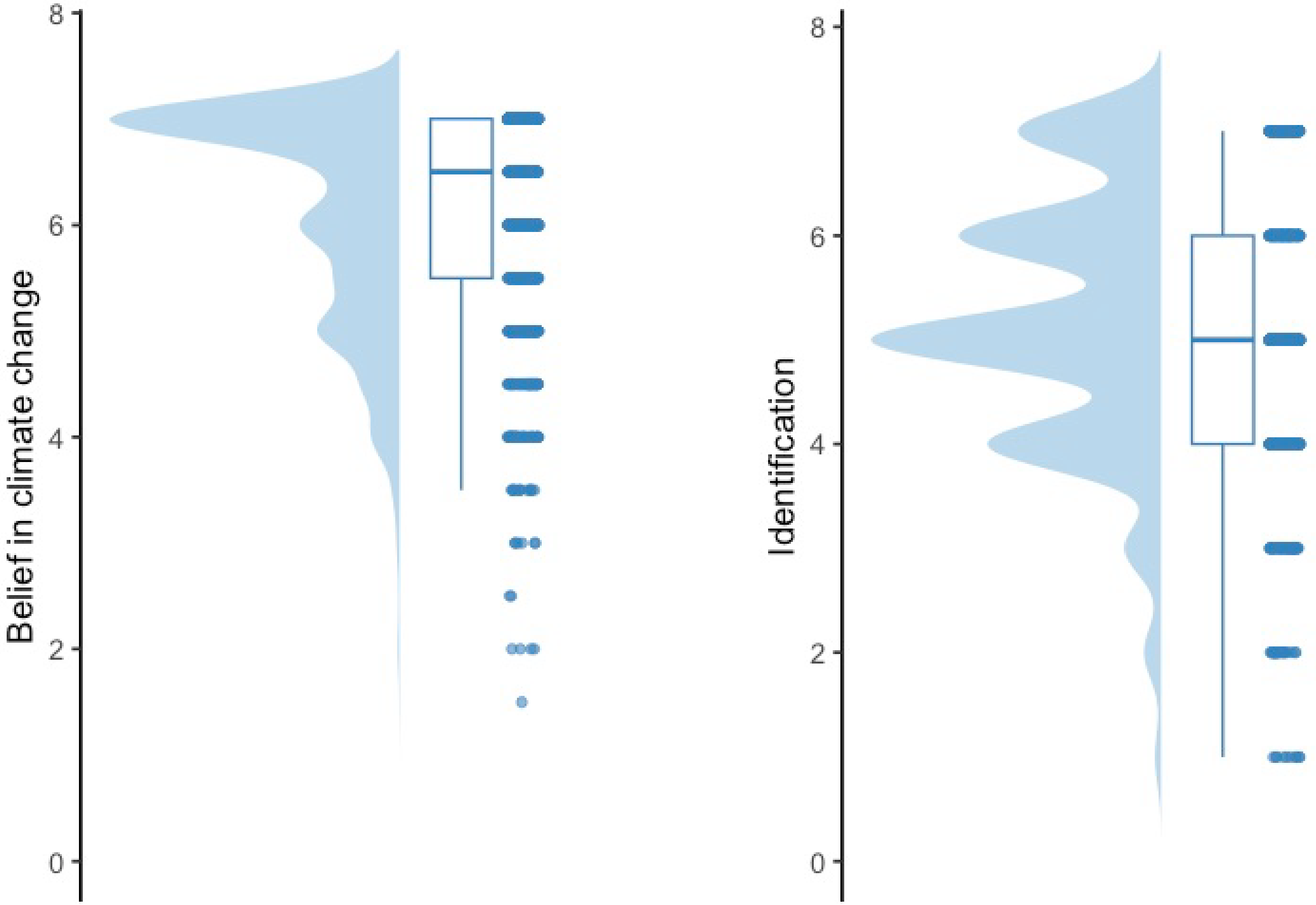
Belief in climate change
8
6
4
2
0
Identification
8
6
4
2
0

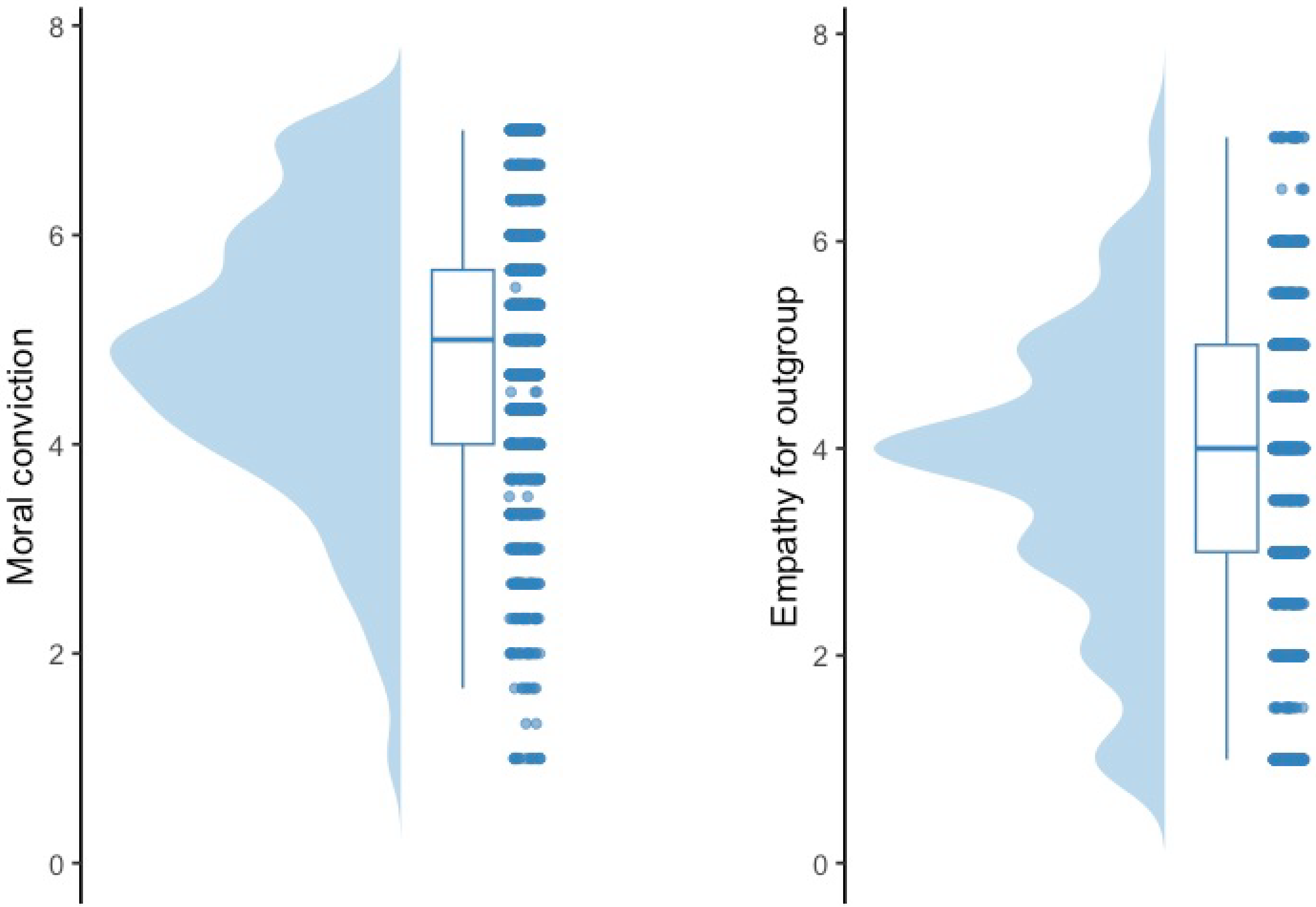
Moral conviction
0
2
4
6
8
Empathy for outgroup
0
2
4
6
8

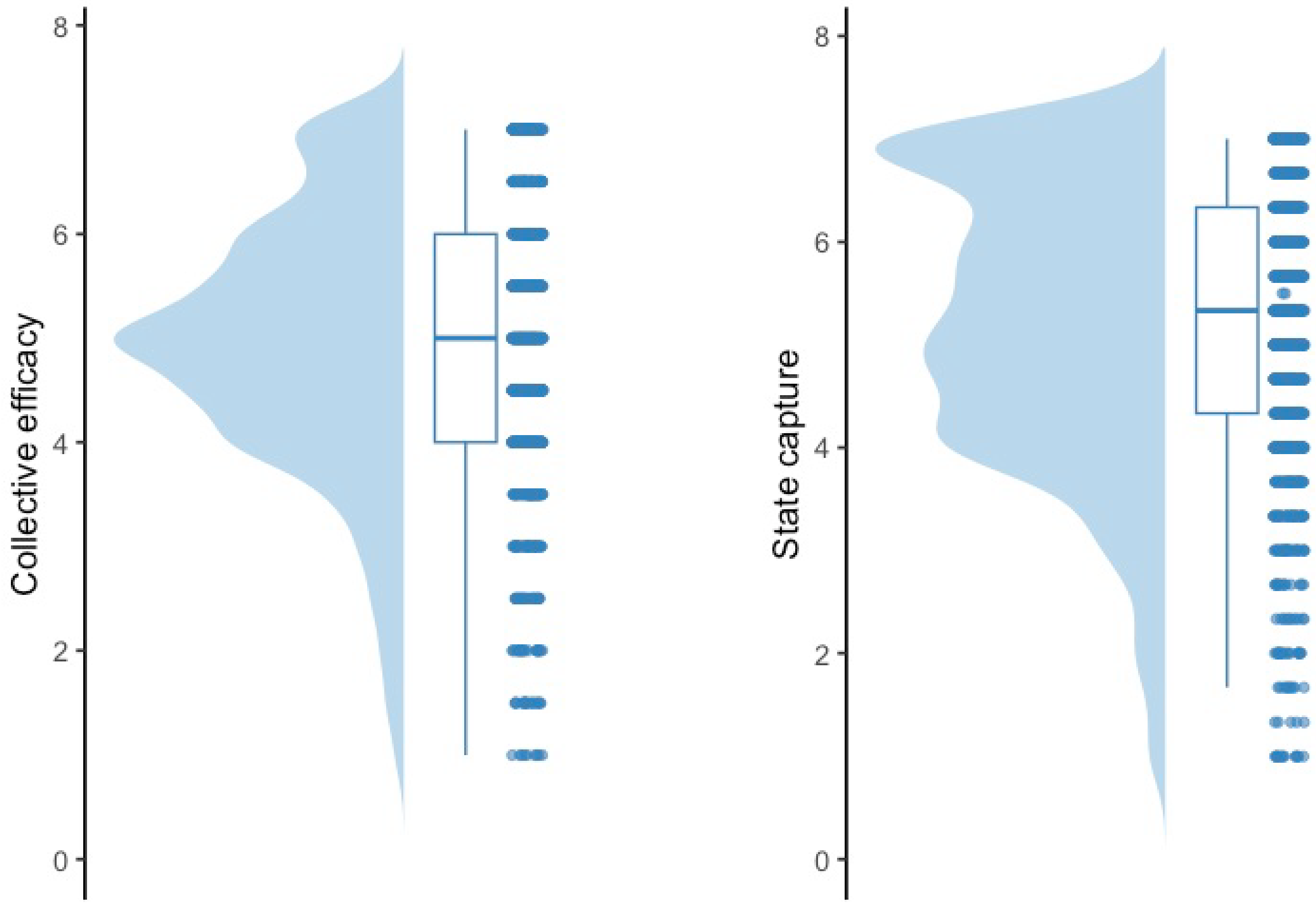
Collective efficacy
State capture
0
2
4
6
8

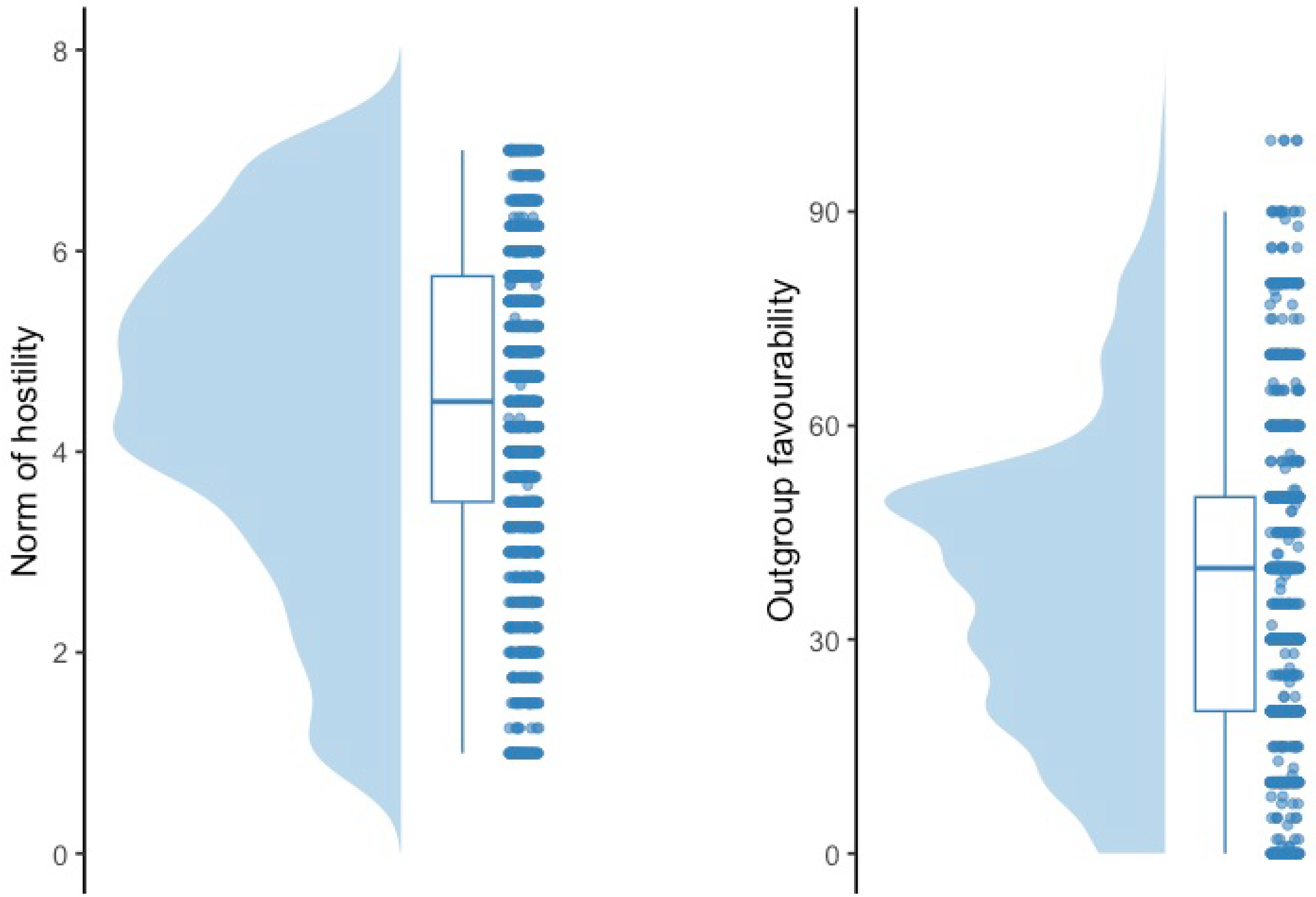
Norm of hostility
0
2
4
6
8
Outgroup favourability
0
30
60
90

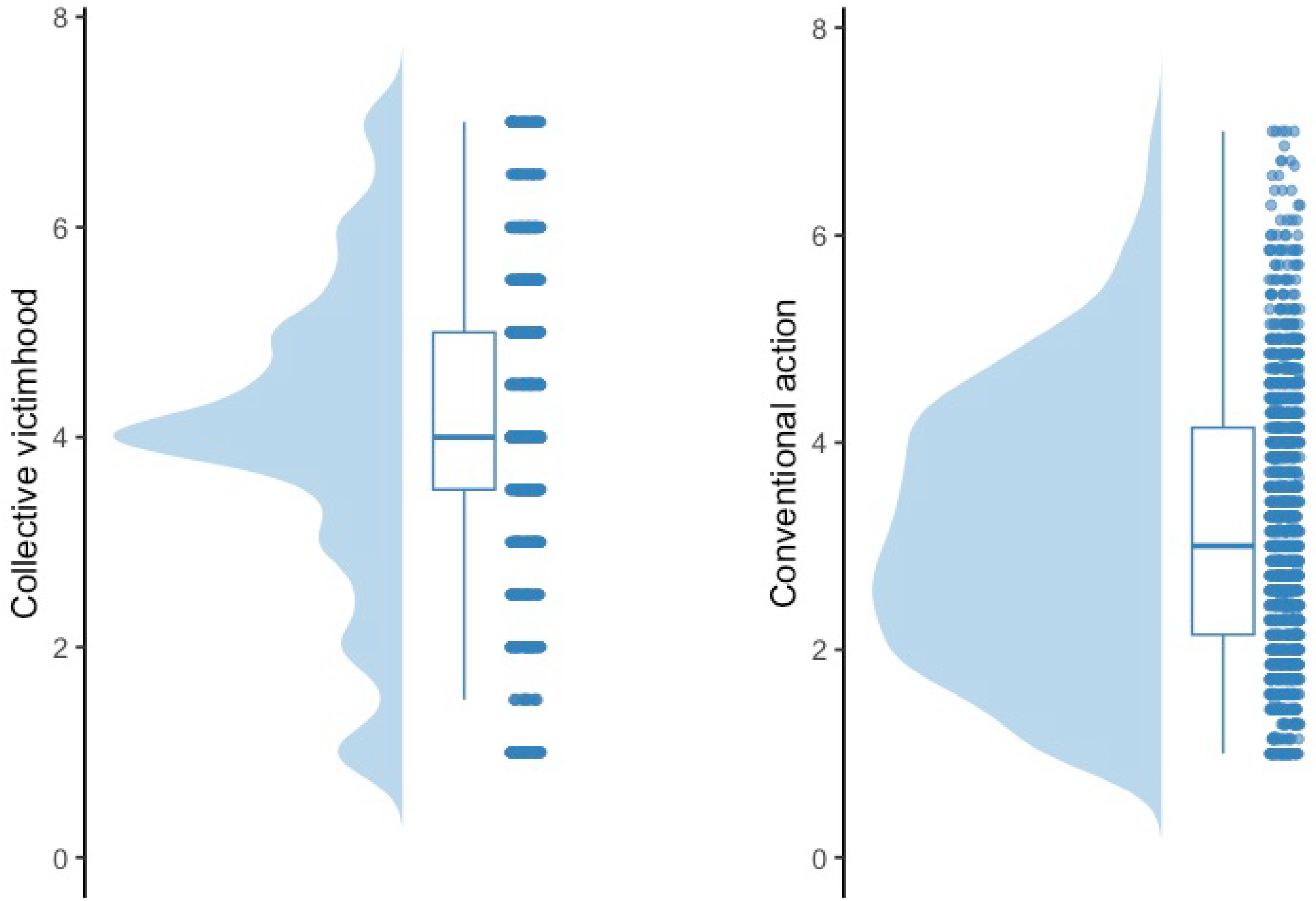

8
6
4
2
0
Collective victimhood
8
6
4
2
0
Conventional action

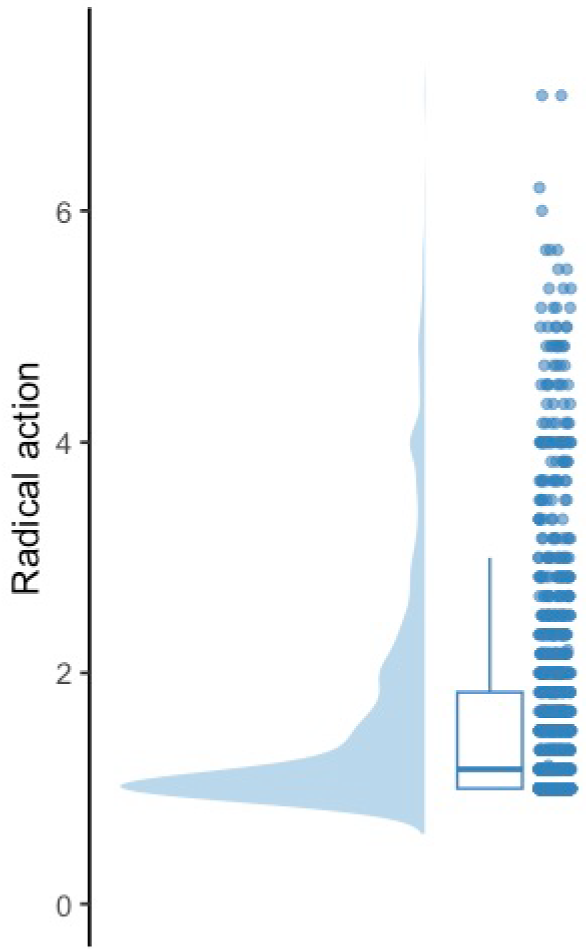
Radical action
0
2
4
6
